# On the Mass Fractal Character of Si-Based Structural Networks in Amorphous Polymer Derived Ceramics

**DOI:** 10.3390/nano5010366

**Published:** 2015-03-17

**Authors:** Sabyasachi Sen, Scarlett Widgeon

**Affiliations:** Department of Materials Science, University of California at Davis, Davis, CA 95616, USA

**Keywords:** polymer derived ceramics, mass fractal, nuclear magnetic resonance (NMR), structural packing

## Abstract

The intermediate-range packing of SiN*_x_*C_4−*x*_ (0 ≤ *x* ≤ 4) tetrahedra in polysilycarbodiimide and polysilazane-derived amorphous SiCN ceramics is investigated using ^29^Si spin-lattice relaxation nuclear magnetic resonance (SLR NMR) spectroscopy. The SiCN network in the polysilylcarbodiimide-derived ceramic consists predominantly of SiN_4_ tetrahedra that are characterized by a 3-dimensional spatial distribution signifying compact packing of such units to form amorphous Si_3_N_4_ clusters. On the other hand, the SiCN network of the polysilazane-derived ceramic is characterized by mixed bonded SiN*_x_*C_4−*x*_ tetrahedra that are inefficiently packed with a mass fractal dimension of *D_f_* ~2.5 that is significantly lower than the embedding Euclidean dimension (*D* = 3). This result unequivocally confirms the hypothesis that the presence of dissimilar atoms, namely, 4-coordinated C and 3-coordinated N, in the nearest neighbor environment of Si along with some exclusion in connectivity between SiC*_x_*N_4−*x*_ tetrahedra with widely different N:C ratios and the absence of bonding between C and N result in steric hindrance to an efficient packing of these structural units. It is noted that similar inefficiencies in packing are observed in polymer-derived amorphous SiOC ceramics as well as in proteins and binary hard sphere systems.

## 1. Introduction

Amorphous polymer-derived ceramics (PDCs) in the Si–C–O and Si–C–N systems constitute technologically promising materials with remarkable thermo-mechanical and chemical properties [1,2,3,4,5,6]. The atomic structure of these materials is a nanocomposite of an amorphous network of corner-shared SiO*_x_*C_4−*x*_ or SiN*_x_*C_4−*x*_ (0 ≤ *x* ≤ 4) tetrahedra and sp^2^ bonded amorphous carbon domains [1,2,3,4]. Development of a fundamental understanding of the structure at various length scales and of the corresponding structure–property relationships are of key importance in designing these nanocomposites for specific engineering applications. A wide variety of spectroscopic, scattering and electron microscopic techniques have been used over the last two decades to extensively investigate the nanostructure of Si-based PDCs [1,2,3,4,5,6,7,8,9,10,11,12,13,14,15,16,17,18]. In particular, results from ^29^Si and ^13^C magic-angle-spinning nuclear magnetic resonance (MAS NMR) spectroscopy have yielded a wealth of information regarding the quantitative speciation of the SiO*_x_*C_4−*x*_ or SiN*_x_*C_4−*x*_ units in the tetrahedral nanodomains and the nature of the bonding in the carbon nanodomains of the PDCs [1,2,3,4]. These results, when taken together, imply a partial spatial segregation of the oxygen (nitrogen)-rich and carbon-rich SiO*_x_*C_4−*x*_ (SiN*_x_*C_4−*x*_) tetrahedral units in the SiOC (SiNC) tetrahedral network. Recent reverse-monte-carlo (RMC) simulation studies of SiOC networks corroborate with this partial clustering scenario [19]. On the other hand, the amorphous carbon nanodomains consist of sp^2^-bonded C, presumably present as turbostratic carbon or as graphene-like sheets [2,3,4,5,6,20,21,22].

Recent ^29^Si NMR spin-lattice relaxation (SLR) studies of SiCO PDCs indicated that the constituent SiO*_x_*C_4−*x*_ tetrahedral units form a network that is packed quite inefficiently such that the spatial distribution of the Si atoms or their mass *M* scales with distance *R* as *M* ~ *R^Df^* with the fractal dimension *D_f_* being substantially smaller (2.0 ≤ *D_f_* ≤ 2.5) than the embedding Euclidean dimension of 3 [3]. The oxygen-rich SiO*_x_*C_4−*x*_ units exhibit *D_f_* = 2.5 while the C-rich units display a lower value of *D_f_* with that for SiC_4_ units being ~2.0. The nearly two-dimensional mass distribution of the SiC_4_ and SiOC_3_ units was conjectured to result from their role in the structure as the linking interfacial “tissue” between the oxygen-rich SiOC network and the amorphous sp^2^-C nanodomains [3]. Additionally and more importantly, it was hypothesized that the mixed coordination of Si, *i.e.*, the presence of both C and O atoms as the nearest neighbors of Si atoms combined with the absence of any carbon-oxygen bonding could have led to steric hindrance in the way of efficient packing of the SiO*_x_*C_4−*x*_ units and consequently a mass-fractal character of the SiOC network [3,19]. This hypothesis can be tested by comparing the packing efficiencies of Si-centered tetrahedra with and without mixed bonding in SiCO or SiCN PDC networks of similar chemical compositions. Here, we report the results of a ^29^Si NMR SLR spectroscopic study of the mass fractal dimension *D* of the spatial distribution of Si atoms in SiCN PDCs with and without mixed bonding, derived from different polymeric precursors. The results provide direct evidence that the mass fractal dimensionality of the spatial distribution of the Si-centered tetrahedra and correspondingly the packing efficiency of the latter at the length scale of a few nanometers is indeed controlled by presence or absence of mixed bonding.

## 2. Experimental Methods

### 2.1. Synthesis

Amorphous SiCN PDCs are typically synthesized by pyrolysis of Si-based preceramic polymers of the general formula –[R^1^R^2^Si−*X*]*_n_*–. These polymers can be polysilylcarbodiimides (*X* = –N=C=N–) or polysilazanes (*X* = NR^3^), where R^1^, R^2^ and R^3^ can be hydrogen, phenyl, methyl, ethyl or vinyl groups [1]. The two SiCN PDC samples used in this study are designated as GM35 and PMVS and were obtained from Drs. Gabriela Mera and Amir Tavakoli, respectively. Details of the synthesis and compositional characterization for these samples can be found in previous publications [4,23]. The GM35 sample was synthesized from a polysilsesquicarbodiimide precursor while the PMVS sample was synthesized from a polymethylvinylsilazane precursor. Pyrolysis for both samples was carried out at 1100 °C in argon atmosphere. Chemical analyses of these two samples as published in previous studies indicate that GM35 has a composition of SiC_3.3_N_2.2_ while that of PMVS is: SiC_1.4_N_0.9_ [4,23].

### 2.2. ^29^Si NMR SLR Spectroscopy

NMR SLR spectroscopy is a unique experimental technique for studying the dimensionality of the spatial distribution of nuclides of interest when the spin-lattice relaxation of the latter occurs primarily through the fluctuation of direct dipolar coupling of the nuclear spins with those of the unpaired electronic spins in the system [24,25]. Such unpaired electronic spins are typically associated with structural defects in the form of dangling bonds or paramagnetic impurities. This mechanism of NMR SLR was shown to be operative for ^29^Si nuclides in SiCO PDCs [3]. The nuclear SLR time *T*_1_ of ^29^Si nuclides in this case is a function of their distance *r* from a paramagnetic center that can be expressed as [25]:

(1) $$\frac{1}{T_{1}}=\frac{A}{r^{6}}$$

where,

(2) $$A=\frac{1}{5}\gamma_{s}^{2}\gamma_{n}^{2}\hbar^{2}S\left(S+1\right)\frac{2\tau_{el}}{1+\omega^{2}\tau_{el}^{2}}$$

In Equation (2), γ_s_ and γ_n_ are the gyromagnetic ratios of the electron and of ^29^Si, respectively. *S* and τ_el_ are the electronic spin quantum number and the characteristic electronic relaxation time, respectively, and ω is the Larmor frequency of ^29^Si nuclides. The temporal evolution of the nuclear magnetization *M_z_*(*t*) after saturation contains contribution from nuclides that are at a distance *r* from the paramagnetic center and is directly proportional to the mass m of the observed ^29^Si nuclides. The mass of a material scales with distance as *m* ~ *r^D^* where *D* is the dimension and describes the spatial distribution of the observed nuclei. Using this relationship and Equation (1), one can obtain:

(3) $$M_{z}\left(t\right)\sim t^{D/6}$$

Hence, for nuclear SLR via dipolar coupling with electronic spins in fixed paramagnetic impurities, measurement of *M_z_*(*t*) after saturation may yield the dimensionality of mass distribution *D* of the nuclides under observation [25]. *D* is typically 3 in three dimensional systems. However, inefficient atomic packing in open structures such as those encountered in foams or gels that are characterized by tenuously connected networks may lead to *D* < 3 [25].

All ^29^Si NMR SLR experiments were carried out using a 7 mm Bruker MAS probe (Bruker Corporation, Karlsruhe, Germany) and a Bruker Avance solid-state spectrometer operating at a Larmor frequency of 99.3 MHz for ^29^Si (11.7 T). Crushed samples were packed in ZrO_2_ rotors and were spun at a rate of 6 kHz. The SLR data were collected using a saturation recovery pulse sequence. A comb of sixteen π/2 (4.0 µs) radio frequency pulses were used to saturate the magnetization. The spectra corresponding to the magnetization recovered following various delay times ranging between 0.01 s and 150 s were collected using a π/2 observation pulse. Depending on the delay time, approximately 600 to 9100 free induction decays were collected and averaged to obtain each ^29^Si spectrum. All ^29^Si spectra were externally referenced to tetramethylsilane.

## 3. Results

**Figure 1:**
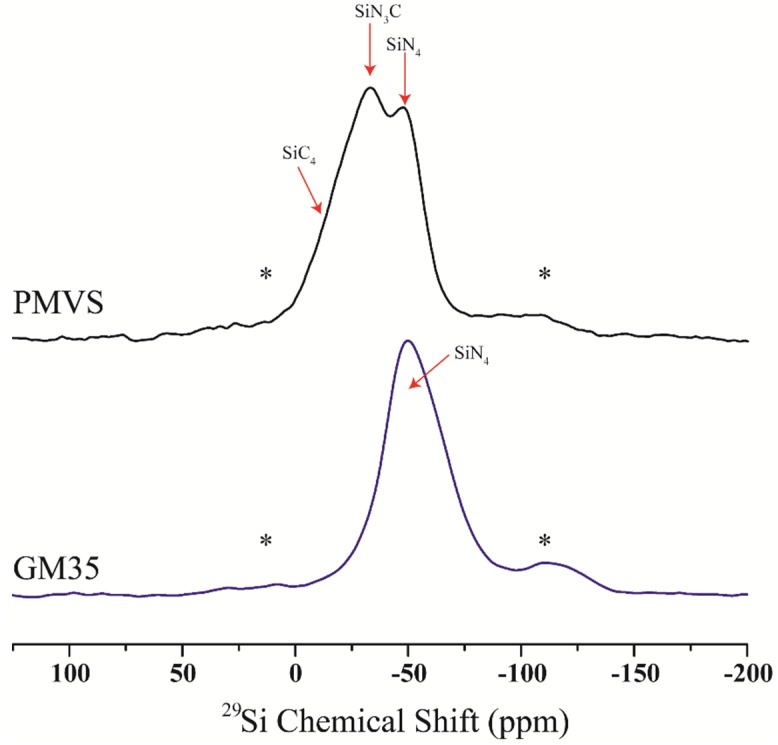
^29^Si MAS nuclear magnetic resonance (NMR) spectra of GM35 (**bottom**) and PMVS (**top**) samples. Spinning sidebands are denoted by asterisks. Structural assignments of the peaks to different Si coordination environments are indicated with arrows.

The ^29^Si MAS NMR spectra of GM35 and PMVS samples are shown in Figure 1. The spectrum of the GM35 sample was already reported in a previous study and shows only one broad resonance at ~−48 ppm corresponding predominantly to Si atoms tetrahedrally coordinated to four N atoms, as in Si_3_N_4_ [4]. The relatively large width of this peak (~32 ppm of full width at half maximum) is consistent with the structural disorder expected in amorphous Si_3_N_4_ domains in these PDCs. The significantly high N/Si value of ~2.2 obtained from the chemical analysis of this sample compared to that expected from the stoichiometry of Si_3_N_4_ (N/Si = 1.33) implies the formation of N–C bonds in the structure. ^15^N NMR spectroscopic results presented in a previous study indeed confirmed that N–C bonds were present most likely at the interface between the Si_3_N_4_ and carbon nanodomains in the GM35 PDC [4]. In contrast with GM35, the ^29^Si spectrum of the PMVS sample (Figure 1) contains multiple peaks corresponding to mixed SiN*_x_*C_4−*x*_ bonding environments. Besides the peak at −49 ppm corresponding to the SiN_4_ tetrahedral environment, this spectrum contains a second peak at −34 ppm and a broad shoulder near –18 ppm which correspond to SiCN_3_ and SiC_4_ structural units, respectively (Figure 1). These results are consistent with the conventional wisdom in the literature that the structure of polysilazane-derived SiCN PDCs is characterized by an amorphous SiCN matrix consisting of mixed bond tetrahedral coordination of Si atoms of the type SiC*_x_*N_4−*x*_ whereas the polysilylcarbodiimide derived SiCN PDCs consist predominantly of SiN_4_ tetrahedral network forming amorphous nanodomains of Si_3_N_4_ [1]. Therefore, the effect of mixed bonding on the tetrahedral packing and mass fractal character of the SiCN network may indeed be investigated by a comparative study between GM35 and PMVS PDCs.

**Figure 2:**
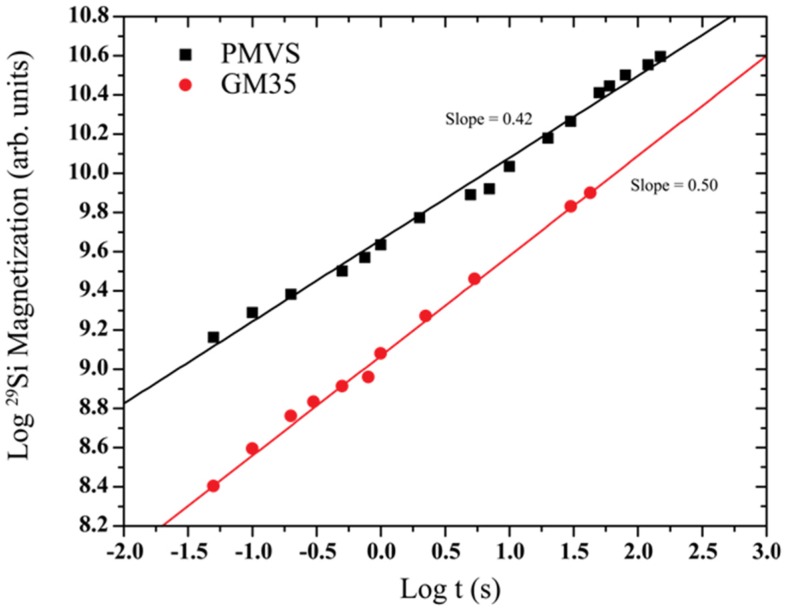
Double logarithmic plots of the recovery of ^29^Si magnetization after saturation, plotted as a function of the delay time for GM35 and PMVS samples. Lines are the linear least-squares fits to the data and represent a power law recovery of magnetization (see text for details) over a time span covering more than three orders of magnitude. The slopes of these lines are reported on the plot.

Recovered magnetizations *M_z_*(*t*) for these ^29^Si spectra in Figure 1, collected at various delay times after saturation, were estimated using the total area under each spectral line shape. The temporal evolution of *M_z_*(*t*) is shown in a log–log plot in Figure 2 for the two PDC samples. The linear dependence of log *M_z_*(*t*) on log t implies a power–law scaling: *M_z_*(*t*) ~ *t*^α^. According to Equation (3), the slope α of the straight line least squares fits to the data in Figure 2 can then be equated to *D*/6. These fits yield α ~0.5 for GM35 while, for PMVS α ~0.42 implying *D_f_* ~3 for the former and ~2.5 for the latter. This difference between the *D_f_* of the spatial distribution of the SiN*_x_*C_4−*x*_ tetrahedra suggests significant differences in the packing efficiency of the tetrahedral units in the networks of the polysilycarbodiimide-derived GM35 and of the polysilazane-derived PMVS ceramics.

## 4. Discussion

As noted above, the ^29^Si MAS NMR spectrum of GM35 in Figure 1 indicates that the tetrahedral network in the structure of this PDC consists primarily of one kind of Si environment, namely, the SiN_4_ tetrahedra. The ^29^Si NMR SLR results indicate that these SiN_4_ tetrahedra are homogeneously and efficiently packed and fill space such that the mass fractal dimension representing their spatial distribution is the same as the embedding Euclidean dimension, *i.e.*, both are 3-dimensional. On the other hand, the tetrahedral network in the structure of PMVS PDC consists of at least three different kinds of SiN*_x_*C_4*−x*_ tetrahedral units: SiN_4_, SiCN_3_ and SiC_4_. The average mass fractal dimension for the spatial distribution of these units is ~2.5 which is less than the embedding dimension, indicating inefficient space filling by the network resulting from the frustrated packing of SiC*_x_*N_4*−x*_ tetrahedra due to the mutual avoidance in connectivity between the SiN_4_ and SiC_4_ tetrahedra and the absence of bonding between C and N atoms within the network. This situation is similar to that reported by us for the mixed-bonded tetrahedral networks in SiCO PDCs where the SiO*_x_*C_4*−x*_ tetrahedra exhibited a mass fractal dimension of ≤2.5 for the various structural units [3]. It is clear from the results of the comparative study presented here that the mass fractal dimensionality lower than the embedding dimension in PMVS originates from the steric constraints in inter-tetrahedral connectivity associated with the coexistence of the 3-fold coordinated N and 4-fold coordinated C atoms in the nearest-neighbor shell of the tetrahedrally coordinated Si atoms. 

**Figure 3:**
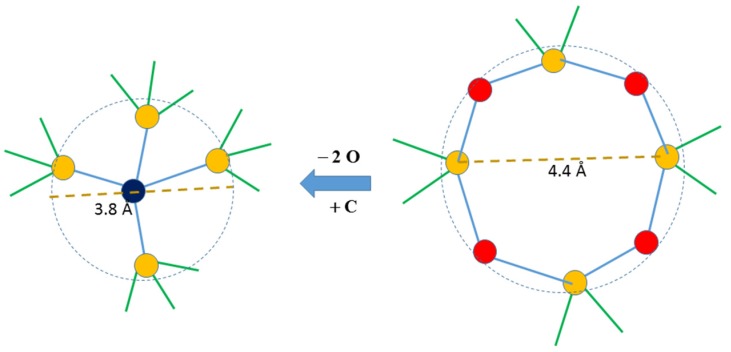
Schematic of the structural transformation of a 4-membered ring of SiO_4_ tetrahedra (right) to a smaller ring of 4 Si atoms (yellow) connected to a central C atom (dark blue) by replacing 2 O atoms (red) in the former with a C atoms, *i.e.*, replacement of a SiO_2_ units with SiC. The dashed lines denote spheres (circles in two-dimension) circumscribing the 4 Si atoms in each case. The diameter of the 4-membered ring is obtained from zeolite crystal structures containing such rings [28]. On the other hand, the diameter of the sphere to the left is twice the typical length of Si–C bonds (1.9 Å) in silicon carbide crystal structure.

**Figure 4:**
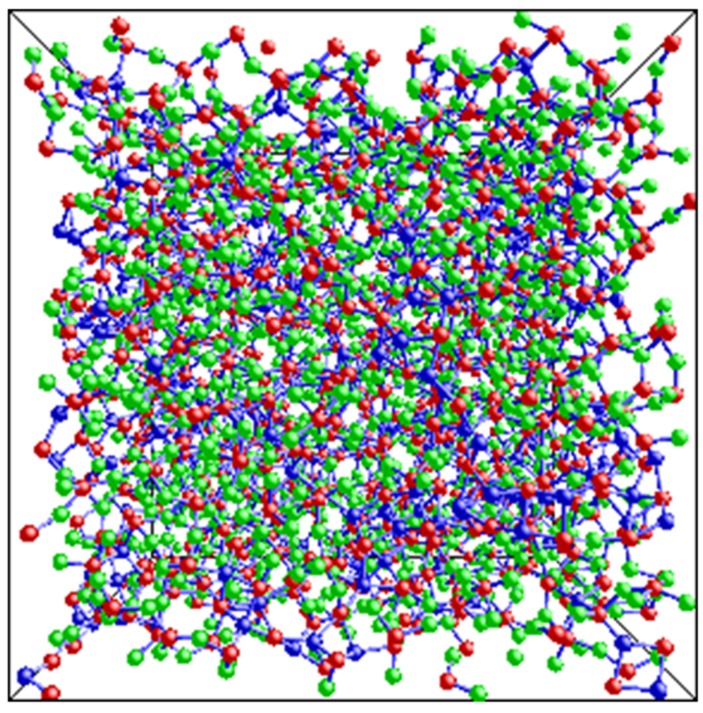
A view of SiOC network obtained in a previous reverse-monte-carlo (RMC) simulation study [19]. Si, O and C atoms are shown in red, green and blue, respectively. Note the inhomogeneous distribution of C atoms implying partial spatial segregation of C containing SiO*_x_*C_4*−x*_ tetrahedra along continuous channel-like regions.

Insights into the origin of the inherent inefficiency in the packing of mixed bonded tetrahedra can be obtained by considering the problem of 3-dimensional packing of binary spheres of similar sizes [26]. This idea is illustrated in Figure 3 by considering the structural transformation of a 4-membered ring of SiO_4_ tetrahedra when SiO_2_ is replaced by SiC to form a SiCO network. Such networks consist predominantly of SiO_4_ and SiO_3_C tetrahedra where the conversion of the former to the latter in Figure 3 results in a smaller ring (diameter of ~3.8 Å) of 4 Si atoms in SiO_3_C tetrahedra around a central C atom, compared to the original 4-membered ring (diameter of ~4.4 Å). This transformation results in a network that can be envisaged as one built by packing two differently sized spheres (differing in size by ~15%). Previous studies have shown that binary spheres differing in size by up to ~30%–35% will pack less efficiently than mono-dispersed spheres [26]. Transformations similar to that shown in Figure 3 can also be visualized, albeit with some difficulty, for larger –Si–O–Si– rings. Inefficiency in tetrahedral packing may not only result in a mass fractal dimension lower than the embedding dimension but eventually may lead to their spatial segregation. Spatial segregation of C containing SiO*_x_*C_4*−x*_ tetrahedra has indeed been observed in the SiCO network in recent RMC simulations (Figure 4) [19]. The limiting value of the mass fractal dimension *D_f_* ~2.5 indicates that the packing of the Si-centered tetrahedra in PDC networks are only as efficient as that of randomly packed spheres near percolation threshold since simulations of the latter was reported to yield *D_f_* ~ 2.5 [27].

Similar inefficiencies in packing have also been reported for a wide variety of protein molecules where the inefficient packing of the constituent amino acids results in an average mass fractal dimension of ~2.5 [29]. Detailed analyses have shown that proteins in their natural configurations fill less than 80% of the volume enclosed by their surface, consistent with a *D_f_* ~2.5, that is significantly less than the embedding dimension [29]. Analysis of *D_f_* in proteins also reveals that its value measured around the center of mass is typically larger than that measured near periphery. It is interesting to compare this result with the *D_f_* values reported in a previous study for the SiO*_x_*C_4*−x*_ units in SiCO networks that indicated a lower *D_f_* (2.0–2.3) for SiC_4_ and SiC_3_O units compared to that (2.3 ≤ *D_f_* ≤ 2.5) for the tetrahedral units richer in oxygen [3]. The lower *D_f_* values for SiC_4_ and SiC_3_O units may then be indicative of the separation of these units near the periphery of the SiCO network. Such separation is also consistent with the constraint that the SiO*_x_*C_4*−x*_ units near the periphery of the network have to be carbon rich to be bonded to the amorphous carbon domains in these PDCs. The mass fractal character of the structural network at the length scale of a few nanometers has been conjectured to be important in controlling the anomalous ionic transport, mechanical properties, structural relaxation (creep) and vibrational localization properties characteristic of the glassy state [27]. It will be interesting to see whether the mass fractal character of the Si containing network in SiCO or SiCN PDCs impart similar anomalies in these unusual amorphous materials.

## 5. Conclusions

^29^Si NMR SLR studies demonstrate that mixed bonded SiO*_x_*C_4*−x*_ or SiN*_x_*C_4*−x*_ (0 ≤ *x* ≤ 4) tetrahedra in SiCO and SiCN PDCs are inefficiently packed, resulting in a mass fractal dimension that is significantly lower than the embedding Euclidean dimension. Such inefficient space filling may result from a combination of different degrees of avoidance in connectivity between various tetrahedra (e.g., complete avoidance between SiN_4_ and SiC_4_ tetrahedra) and the absence of bonding between C and N or O atoms in the network. Proteins and binary hard sphere systems display similar inefficiencies in packing. Further studies are required to investigate how the mass fractal character of the tetrahedral network may influence the physical properties of PDCs.
